# New Multifeature Information Health Index (MIHI) Based on a Quasi-Orthogonal Sparse Algorithm for Bearing Degradation Monitoring

**DOI:** 10.1155/2021/2221702

**Published:** 2021-08-03

**Authors:** Xiao Zhang, Tengyi Peng, Shilong Sun, Yu Zhou

**Affiliations:** ^1^College of Computer Science, South-Central University for Nationalities, Wuhan 430074, China; ^2^School of Mechanical Engineering and Automation, Harbin Institute of Technology, Shenzhen 518055, China; ^3^College of Computer Science and Software Engineering, Shenzhen University, Shenzhen, China

## Abstract

Data-driven intelligent prognostic health management (PHM) systems have been widely investigated in the area of defective bearing signals. These systems can provide precise information on condition monitoring and diagnosis. However, existing PHM systems cannot identify the accurate degradation trend and the current fault types simultaneously. Given that different fault types have various effects on the mechanical system, the corresponding maintenance strategies also vary. Then, choosing the appropriate maintenance strategy according to the future fault type can reduce the maintenance cost of the equipment operation. Therefore, a multifeature information health index (MIHI) must be developed to trace various bearing degradation trends with various types of faults simultaneously. This paper reports a new quasi-orthogonal sparse project algorithm that can mutually convert the degraded processing feature vector sets (such as spectrum) for each type of fault to orthogonal approximate spatial straight lines. The algorithm builds a MIHI through the spectrum of current state measured points. The MIHI is then transformed by a quasi-orthogonal sparse project algorithm to trace the various bearing degradation trends and recognize the fault type simultaneously. The case study of bearing degradation data demonstrates that this approach is effective in assessing the various degradation trends of different fault types.

## 1. Introduction

With the progress of science and technology, building an effective intelligent prognostic health management (PHM) system has become necessary for some critical components. Engineers deploy various types of sensors to detect the health conditions of a single component. However, how to deal with sensor information effectively and thus assist the intelligent health management of equipment are yet to be extensively researched. Remaining useful life (RUL) estimation is one of the key factors in asset condition-based maintenance, prognostics, and health management [1]. The basic principle of RUL estimation is how to construct the health index [2]. An appropriate health index can effectively improve the accuracy of RUL prediction. To detect the incipient faults and offer effective information to the RUL estimation and prognostics model, the health index plays an important role as a bridge connecting the sensor measured signal with the assets' health condition prognostics. Some statistical indicators, such as root mean square (RMS) and the kurtosis of the measured vibration signal [3], are used to indicate the assets' conditions for RUL estimation and prognostics. Moreover, some studies utilize time-frequency features, such as waveform entropy [4]. Many dimensionality reduction methods have been favored by researchers, for example, kernel principal component analysis is used in [5] and isometric feature mapping is used in [6]. In recent studies, researchers have developed some effective statistical indicators, i.e., using multiscale fuzzy entropy [7], Kullback–Leibler divergence combined with Gaussian process regression [8], and a neural network to conduct a health index to monitor the equipment condition directly [9, 10].

The above health index building methods are used in prognostics, and they mostly map the information from various sensor signals or feature extractors into an indicator full of information. This process is called data fusion, which has three categories: feature-level fusion, decision-level fusion, and data-level fusion [11]. Feature-level fusion methods rely on prior knowledge of the degradation mechanism and physical models to analyse the input data. Goebel [12] utilized a feature-level fusion model that consisted of principal component analysis, filtering, smoothing, normalization, and log-transformation methods to feed information to the RUL estimation model and predict the breakage of paper webs in a paper-making machine at the wet end. Ma [13] reported a multiple-view feature fusion to predict lithium-ion battery RUL. Decision-level methods fuse high-level decisions made based on individual sensor data and do not rely on raw signal feature extraction. Niu et al. [14] combined the advantages of wavelet analysis on transient signals and decision-level fusion techniques to improve the accuracy of fault diagnosis. Wei [15] proposed a decision-level data fusion method to map the individual sensor signal into reliable data to improve the ability of the quality control system in additive manufacturing and RUL estimation of aircraft engines.

In contrast to feature- and decision-level fusion methods that focus on the physical meaning, data-level fusion methods pay attention to mining the embedding feature suitable to the task from the raw data. In RUL prediction and condition monitoring fields, data-level fusion methods always build the health index based on some properties that the researchers hope can improve the RUL estimation ability. Data-level data fusion methods are suitable for dealing with complex system situations because these systems hardly build an effective model to fuse various signals, but data-level can monitor the machinery system state according to the requirements of monitoring task, which has a stronger versatility. In summary, the data-level fusion method is concentrated on the property of required tasks. Some scholars have focused on investigations of properties on the health index. Two properties of the health index are proposed by Liu [16]. First, it must have good monotonicity; second, the variance of the failure threshold of multiple experiments must be minimized. Chehade [17] proposed another proposition: separability. They thought the greater the difference of the health index between two observations, the more validated and reliable the health index. In [2], the development of the health index focused on monotonicity and separability, and it converted the transform matrix solving the problem to a convex optimization process. Yan [18] focused on locating informative frequency bands, determining the optimal fault frequency band for health indicating. Some researchers combined the health index building with statistical analysis. For example, Kim [19] proposed a linear multisensor information fusion method to build a health index and derive the best linear unbiased estimator of the fusion coefficients. At the same time, some researchers studied the methods based on the Kalman filter to fuse the sensor signals (e.g., Markov model [20]). To deal with the sensor selection problem from the multiple sensors situation, Liu [21] studied the perspective of the quality of the signal provided by sensors and proposed a signal-to-noise ratiometric method to combine various sensor signals, develop a health index, and monitor asset degradation. However, from the current study of bearing health condition monitoring, the sparsity used in building a health index is rarely considered. The lack of sparse terms leads to overfitting and reduces the generalization ability of the health index. In data-driven model building, the sparse penalty term is also used to make models have additional reusability and avoid overfitting. Sparse models built via lasso (L1-normalization) can achieve interpretable feature selection [22], and lasso has been widely investigated in the fields of biology and medicine. For some features with adjacent relevance, the fused lasso is proposed to make feature weights sparse and smooth [23].

Given that the high-dimensional feature is difficult to calculate and observe, some scholars use the health index to monitor equipment's operating condition intuitively and conduct prognostic health management. The degradation trend of different types of faults varies. Meanwhile, information on the current faults' evolution direction can also further improve the accuracy of RUL estimation. Therefore, this study aims to provide a method for constructing a health index that can indicate various fault type degradation trends from the fusion of the features at the same time. The contributions of this study can be summarized as follows:

1. This work proposes an orthogonal proposition of developing a health index. This proposition focuses on expanding the discrepancy of different fault type degradations, which is the basic idea of the multifeature information health index (MIHI).
2. The MIHI uses a quasi-orthogonal sparse projection algorithm to convert the spectral features of the current measurement point into a low-dimensional vector. This low-dimensional vector can simultaneously represent the bearing degradation trend of multiple types of faults.
3. The weight sparse and the weight difference sparse are added to MIHI to build an objection function and improve the general applicability.
4. The optimization problem expression of the proposed quasi-orthogonal sparse project algorithm is a nonconvex function with constraint. The fast-solving algorithm based on iteration is given.

## 2. Proposed Quasi-Orthogonal Sparse Project Algorithm

In this section, a quasi-orthogonal sparse project algorithm is developed to transfer the spectrum feature to a low-dimensions' vector health index for bearing degradation monitoring. To efficiently trace various fault type degradation trends, the project algorithm should satisfy the five properties:

**Property 1 .**

Monotonicity: once an initial fault occurs, the trend of the degradation signals should be monotonic [16].

**Property 2 .**

Sensitivity: the health index should be sensitive to bearing components that generate abnormal defects, that is, the health index can separate the normal and abnormal health conditions of bearings [24].

**Property 3 .**

Orthogonality: to indicate the various types of bearing fault effectively, the health index is built as a vector, and the size of the health index vector should be the same as the number of the fault types. To avoid confusion of the fault types in the health index, each dimension of the health index should be orthogonal.

**Property 4 .**

Weight sparsity: to prevent overfitting, the weight of the features with low correlation to Properties 1–3 should be small. We also hope that the weights of these features can be set as 0 to achieve feature selection.

**Property 5 .**

Weight difference sparsity: under rotating machinery operating conditions with load, working frequency has some small fluctuations (especially, bearing ball pass frequency). The weights' difference sparsity helps the projection matrix to become more flexible and deal with the fluctuating frequencies. The principle of MIHI is extracting the spectrum characteristics, which can track the degradation trend and distinguish different fault types from degradation datasets of various faults. Given that the bearing fault impulse signal is a pseudocyclization signal (the period of impulse random fluctuates around a mean value), the characteristic frequency of the fault fluctuates in a small interval. The fused lasso sparse term makes HIMI's weight matrix focus on the frequency band around the mean characteristic frequency rather than the signal characteristic frequency. Hence, this term can prevent overfitting.

**Figure 1:**
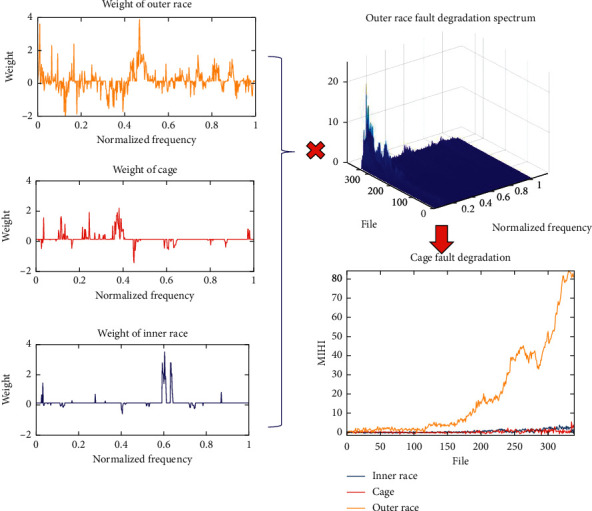
Illustrative example for building MIHI from a spectrum.

In summary, the quasi-orthogonal sparse project algorithm for building a sparse multi-information feature health index is expected to detect bearing fault types and evaluate the bearing degradation process monotonically and sensitively at the same time. The proposed quasi-orthogonal project algorithm is based on the traditional linear fitting method, and the equation is defined as follows:

(1) HIΜΙi=W×f,i

where **W** ∈ *ℝ*^*k*×*m*^ is the projection matrix, *m* denotes the size of the spectrum feature **f**_*i*_ of the *i*th observation, *k* is the fault type number, and **HI**ΜΙ_*i*_ ∈ *ℝ*^*k*×1^.  Figure 1 shows the example of equation (1).

To deal with various fault type monitoring tasks, the proposed algorithm needs different types of fault degradation datasets as prior knowledge. We denote the *j*th fault type degradation data as **F**_*j*_ ∈ *ℝ*^*m*×*n*_*j*_^, where *n*_*j*_ denotes the number of observation epochs of the *j*th fault type degradation. We also denote the *i*th observation epoch feature vector of the *j*th fault type degradation data as **f**_*j*,*i*_.

In addition, the projection matrix can be denoted as **W**=[**w**_1_^*T*^, **w**_2_^*T*^,…,**w**_*k*_^*T*^]^*T*^, and the weights in this projection matrix **W** need to be calculated row by row. The solving process of the *j*th row of the projection matrix **w**_*j*_ can be described as an optimization problem, and the formula of this optimization is provided as follows:

(2) $$\begin{array}{c} \underset{w_{j}}{\text{maximize}}\left\{D_{\text{sensitive}}^{2}\left(w_{j}\right)-D_{\text{orthogonality}}^{2}\left(w_{j}\right)-\alpha D_{\text{monotonousness}}^{2}\left(w_{j}\right)-\beta \overset{m}{\underset{q=1}{\sum }}\left|w_{j,q}\right|-\gamma \overset{m}{\underset{q=2}{\sum }}\left|w_{j,q}-w_{j,q-1}\right|\;\right\}, \end{array}$$

where *α* is defined to balance sensitivity and monotonicity (corresponding to Property 1 and 2), *β* is the sparsity penalty parameter (corresponding to Property 4), and *γ* is the weight difference sparsity penalty parameter (corresponding to Property 5). *μ*_*j*_ denotes the mean vector of the normal state observation matrix **N**_*j*_=[**f**_*j*,1_, **f**_*j*,2_,…, **f**_*j*,*n*_nor__], and *n*_nor_ is the number of the first few observation epochs (generally, the bearing signals can be assumed as normal signals in the first few observation epochs), and *n*_nor_ corresponds to Property 3.

Then, *D*_sensitive_^2^(**w**_*j*_) can be calculated by

(3) $$\begin{array}{c} D_{\text{sensitive}}^{2}\left(w_{j}\right)=\overset{n_{j}}{\underset{i=1}{\sum }}{\left\|w_{j}\times \left(f_{j,i}-\mu_{j}\right)\right\|}_{2}^{2}. \end{array}$$

To ensure that the health index can sensitively monitor the abnormal condition, *D*_sensitive_^2^(**w**_*j*_) needs to be as large as possible.

*D*_monotonousness_^2^(**w**_*j*_) corresponds to Property 1. To reduce computational complexity, health index monotonicity relies on *D*_sensitive_^2^(**w**_*j*_) and *D*_monotonousness_^2^(**w**_*j*_). *D*_monotonousness_^2^(**w**_*j*_) is used to evaluate the stability of the different health indexes of two adjacent observation epochs, and it can be calculated by

(4) $$\begin{array}{c} D_{\text{monotonousness}}^{2}\left(w_{j}\right)=\overset{n_{j}}{\underset{i=2}{\sum }}{\left\|w_{j}\times \left(f_{j,i}-f_{j,i-1}\right)\right\|}_{2}^{2}, \end{array}$$

where *D*_monotonousness_^2^(**w**_*j*_) can only assess the stability but not the monotonicity of the health index. However, enlarging *D*_sensitive_^2^(**w**_*j*_) and shrinking *D*_monotonousness_^2^(**w**_*j*_) simultaneously can effectively approximate the realization of monotonicity. To achieve orthogonality, we need to reduce the health index difference between the other fault type data and the current fault type health state. *D*_orthogonality_^2^(**w**_*j*_) is calculated by

(5) $$\begin{array}{c} D_{\text{orthogonality}}^{2}\left(w_{j}\right)=\overset{j-1}{\underset{v=1}{\sum }}\overset{n_{j}}{\underset{i=1}{\sum }}{\left\|w_{j}\times \left(f_{v,i}-\mu_{j}\right)\right\|}_{2}^{2}+\overset{k}{\underset{v=j+1}{\sum }}\overset{n_{j}}{\underset{i=1}{\sum }}{\left\|w_{j}\times \left(f_{v,i}-\mu_{j}\right)\right\|}_{2}^{2}+\overset{n_{\text{nor}}}{\underset{i=1}{\sum }}{\left\|w_{j}\times \left(f_{j,i}-\mu_{j}\right)\right\|}_{2}^{2}, \end{array}$$

where *D*_orthogonality_^2^(**w**_*j*_) assesses the health index value difference of other fault types of degradation data with the *j*th fault type normal condition data. To realize orthogonality, *D*_orthogonality_^2^(**w**_*j*_) needs to be as small as possible.

To reduce computation complexity, *D*_sensitive_^2^(**w**_*j*_), *D*_monotonousness_^2^(**w**_*j*_), and *D*_orthogonality_^2^(**w**_*j*_) are simplified as follows:

(6) $$\begin{array}{c} D_{\text{sensitive}}^{2}\left(w_{j}\right)=w_{j}\times \left\{\overset{k}{\underset{i=1}{\sum }}\overset{n_{i}}{\underset{j=1}{\sum }}\left[\left(f_{i,j}-\mu_{j}\right)\times {\left(f_{i,j}-\mu_{j}\right)}^{T}\right]\right\}\times w_{j}^{T},\\ =w_{j}\times S_{\text{sensitive}}^{j}\times w_{j}^{T}, \end{array}$$

(7) $$\begin{array}{c} D_{\text{monotonousness}}^{2}\left(w_{j}\right)=w_{j}\times \left\{\overset{n_{j}}{\underset{j=2}{\sum }}\left[\left(f_{j,i}-f_{j,i-1}\right)\times {\left(f_{j,i}-f_{j,i-1}\right)}^{T}\right]\right\}\times w_{j}^{T},\\ =w_{j}\times S_{\text{monotonousness}}^{j}\times w_{j}^{T},\\ =w_{j}\times S_{\text{orthogonality}}^{j}\times w_{j}^{T}. \end{array}$$

(8) $$\begin{array}{c} D_{\text{orthogonality}}^{2}\left(w_{j}\right)=w_{j}\times \left\{\overset{j-1}{\underset{v=1}{\sum }}\overset{n_{j}}{\underset{i=1}{\sum }}\left[\left(f_{v,i}-\mu_{j}\right)\times {\left(f_{v,i}-\mu_{j}\right)}^{T}\right]+\overset{k}{\underset{v=j+1}{\sum }}\overset{n_{j}}{\underset{i=1}{\sum }}\left[\left(f_{v,i}-\mu_{j}\right)\times {\left(f_{v,i}-\mu_{j}\right)}^{T}\right]+\overset{n_{\text{nor}}}{\underset{i=1}{\sum }}\left[\left(f_{j,i}-\mu_{j}\right)\times {\left(f_{j,i}-\mu_{j}\right)}^{T}\right]\right\}\times w_{j}^{T},\\ =w_{j}\times S_{\text{orthogonality}}^{j}\times w_{j}^{T}. \end{array}$$

Substituting equations (6)–(8) into equation (2), referring to the calculation from Fisher's discriminant ratio, and then rewriting equation (2), we obtain

(9) $$\begin{array}{c} \underset{w_{j}}{\text{maximize}}\left\{w_{j}S_{\text{sensitive}}^{j}w_{j}^{T}-\beta_{j}\overset{m}{\underset{q=1}{\sum }}\left|\hat{\sigma_{j,q}}w_{j,q}\right|-\gamma_{j}\overset{m}{\underset{q=2}{\sum }}\left|\hat{\sigma_{j,q}}w_{j,q}-\hat{\sigma_{j,q-1}}w_{j,q-1}\right|\;\right\},\\ \text{subject to}w_{j}S_{\text{subject}}^{j}w_{j}^{T}\leq 1, \end{array}$$

where **S**_subject_^*j*^=**S**_orthogonality_^*j*^+*α*^2^**S**_monotonousness_^*j*^. $\hat{\sigma_{j,q}}$ gives more penalty to the features that cause MIHI monotonicity and orthogonality showing fluctuations. $\hat{\sigma_{j}}=\left(\hat{\sigma_{j,1}},\hat{\sigma_{j,2}},\ldots ,\hat{\sigma_{j,m}}\right)$ is calculated by the following formula:

(10) $$\begin{array}{c} \hat{\sigma_{j,q}}=\text{std}\left(O_{j}\left(:,q\right)\right)+\alpha^{2}\times \text{std}\left(P_{j}\left(:,q\right)\right), \end{array}$$

where **O**_*j*_=[**F**_1_, **F**_2_,…, **F**_*j*−1_, Ν_*j*_, **F**_*j*+1_,…, **F**_*k*_] and **P**_*j*_=[**f**_*j*,2_ − **f**_*j*,1_, **f**_*j*,2_ − **f**_*j*,1_,…, **f**_*j*,*n*_*j*__ − **f**_*j*,*n*_*j*_−1_]. Moreover, in equation (9), *β*_*j*_=*β*‖**S**_subject_^−1/2^**S**_sensitive_**S**_subject_^−1/2^‖ and *γ*_*j*_=*γ*‖**S**_subject_^−1/2^**S**_sensitive_**S**_subject_^−1/2^‖, where ‖.‖ indicates the largest eigenvalue.

Lastly, according to the health index of *j*th fault type value changing trend is positive or negative, multiplying 1 or -1 with **w**_*j*_ to ensure the health index has an increasing trend.

## 3. Solving Process

### 3.1. Weight Matrix Solving

**Figure 2:**
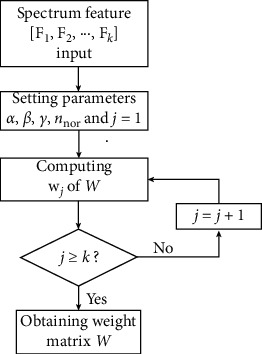
Solving weight matrix of the quasi-orthogonal project algorithm.

Figure 2 shows the solving flowchart of the proposed quasi-orthogonal sparse project algorithm. First, the historical data of various types of faults are reprocessed, transferring all observation signals to the spectrum feature [**F**_1_, **F**_2_,…, **F**_*k*_]. Second, the parameters *α*, *β*, *γ*, and *n*_nor_ are set, and each row of the weight matrix **W** is solved in turns.

### 3.2. Solution Detail of the Weight Vector

In this section, the solution of equation (10) is provided. In general, equation (9) cannot be solved using tools from convex optimization. According to [25], we need to use a minimization-maximization algorithm to rewrite it. The first step is to construct an iterative from equation (9):

(11) $$\begin{array}{c} \underset{d}{\text{minimize}}\left\{dS_{\text{subject}}^{j}d^{T}-2dS_{\text{sensitive}}^{j}{\left(w_{j}^{\left(h\right)}\right)}^{T}+\beta_{j}\overset{m}{\underset{q=1}{\sum }}\left|\hat{\sigma_{j,q}}d_{j,q}\right|+\gamma_{j}\overset{m}{\underset{q=2}{\sum }}\left|\hat{\sigma_{j,q}}d_{j,q}-\hat{\sigma_{j,q-1}}d_{j,q-1}\right|\right\}. \end{array}$$

From the result $\overset{⌢}{d}$ of equation (11), we can obtain $w_{j}^{\left(h+1\right)}=\overset{⌢}{d}/\sqrt{\overset{⌢}{d}S_{\text{subject}}^{j}{\overset{⌢}{d}}^{T}}$ (if $\overset{⌢}{d}=0$, **w**^(*h*)^=0).

The second step is to build a transfer matrix **R** ∈ *ℝ*^(*m* − 1)×*m*^:

(12) $$\begin{array}{c} R=\left(\begin{array}{ccccc} -1 & 1 & 0 & \ldots & 0\\ 0 & -1 & 1 & \ddots & \vdots \\ \vdots & \ddots & \ddots & \ddots & 0\\ 0 & \ldots & 0 & -1 & 1 \end{array}\right)\left(\begin{array}{cccc} \hat{\sigma_{j,1}} & 0 & \ldots & 0\\ 0 & \hat{\sigma_{j,2}} & \ddots & \vdots \\ \vdots & \ddots & \ddots & 0\\ 0 & \ldots & 0 & \hat{\sigma_{j,m}} \end{array}\right). \end{array}$$

Substituting equation (12) into equation (11), we obtain

(13) $$\begin{array}{c} \underset{d}{\text{minimize}}\left\{dS_{\text{subject}}^{j}d^{T}-2dS_{\text{sensitive}}^{j}{\left(w^{\left(h\right)}\right)}^{T}+\beta_{j}{\left\|\hat{\sigma_{j}}\circ d\right\|}_{1}+\gamma_{j}{\left\|b\right\|}_{1}\right\},\\ \text{subject to}Rd^{T}=b, \end{array}$$

where ∘ denotes Hadamard product. Then, the augmented Lagrange function of equation (13) is built as follows:

(14) $$\begin{array}{c} \underset{d}{\text{minimize}}\left\{{\left\|Ad^{T}-c\right\|}_{2}^{2}+\beta_{j}{\left\|\hat{\sigma_{j}}\circ d\right\|}_{1}+\gamma_{j}{\left\|b\right\|}_{1}-e\left(Rd^{T}-b\right)+\frac{\eta }{2}{\left\|Rd^{T}-b\right\|}_{2}^{2}\right\}, \end{array}$$

where $A=Q\times \sqrt{G}$, **Q** and **G** satisfy **S**_subject_^*j*^=**Q**^*T*^**GQ**, **G** is a diagonal matrix, and **c**=**w**_*j*_^(*h* − 1)^ × **S**_sensitive_^*j*^ × inv(**A**). By using the linearized alternating direction method [26], iterative augmented Lagrange function equation (14) can be rewritten as three subequations:

(15) $$\begin{array}{c} \left\{\begin{array}{c} d^{\left(z+1\right)}=\underset{d}{\text{minimize}}\left\{\beta_{j}{\left\|\hat{\sigma_{j}}\circ d\right\|}_{1}+\frac{l}{2}{\left\|d-d^{\left(z\right)}+\frac{{\hat{X}}^{T}\left(\hat{X}{\left(d^{\left(z\right)}\right)}^{T}-{\hat{y}}^{\left(z\right)}\right)}{l}\right\|}_{2}^{2}\right\},\\ b^{\left(z+1\right)}=\underset{b}{\text{minimize}}\left\{\gamma_{j}{\left\|b\right\|}_{1}+\frac{\eta }{2}{\left\|b-R{\left(d^{\left(z+1\right)}\right)}^{T}+\frac{e^{\left(z\right)}}{\eta }\right\|}_{2}^{2}\right\},\\ e^{\left(z+1\right)}=e^{\left(z\right)}-\eta \left(R{\left(d^{\left(z+1\right)}\right)}^{T}-b^{\left(z+1\right)}\right), \end{array}\right. \end{array}$$

where $\hat{X}={\left({\left(\sqrt{2}A\right)}^{T},{\left(\sqrt{\eta }R\right)}^{T}\right)}^{T}$, ${\hat{y}}^{\left(w-1\right)}={\left({\left(\sqrt{2}c\right)}^{T},{\left(\sqrt{\eta }\left(b^{\left(w-1\right)}+e^{\left(w-1\right)}/\eta \right)\right)}^{T}\right)}^{T}$, and *l* is the approximation parameter. To ensure the convergence of equation (15), *l* > *ρ*(**A**^*T*^**A**+*η ***R**^*T*^**R**). Using a soft-threshold algorithm can obtain the closed-form solution of equation (15):

(16) $$\begin{array}{c} \left\{\begin{array}{c} d^{\left(z+1\right)}=\text{shrinkage}\left(\left(d^{\left(z\right)}-\frac{{\hat{X}}^{T}\left(\hat{X}{\left(d^{\left(z\right)}\right)}^{T}-{\hat{y}}^{\left(z\right)}\right)}{l}\right),\left(\left(\frac{\beta_{j}}{l}\right)\hat{\sigma_{j}}\right)\right),\\ b^{\left(z+1\right)}=\text{shrinkage}\left(\left(R{\left(d^{\left(z+1\right)}\right)}^{T}-\frac{e^{\left(z\right)}}{\eta }\right),\left(\frac{\gamma_{j}}{\eta }\right)\right),\\ e^{\left(z+1\right)}=e^{\left(z\right)}-\eta \left(R{\left(d^{\left(z+1\right)}\right)}^{T}-b^{\left(z+1\right)}\right), \end{array}\right. \end{array}$$

where shrinkage(**v**_1_, **v**_2_)≜sign(**v**_1_) · max{0, |**v**_1_| − **v**_2_}.

Following the above deduction, the solving process of equation (9) can be summarized as follows (Algorithm 1):

## 4. Case Study

### 4.1. Data of Illustrative Example

In this illustrative example, the bearing run-to-failure data are studied. Bearing fault datasets have three types: inner race fault, cage, and outer race fault. The run-to-failure data from XJTU are measured under the condition of 11 kN load, 2250 rpm speed, and 25.6 kHz sampling frequency. The data files that come from bearing 2_1, bearing 2_3, and bearing 2_5 in reference.  Table 1 is used to calculate the weight matrix.

In each observation epoch, the length of the signal that the sensor collected is more than 20000; thus, the size of the spectrum generated via FFT transform can be more than 10000. To reduce computational complexity, in this case, FFT only generates 512 dimensions' spectrums as the quasi-orthogonal sparse project algorithm input data.

### 4.2. Results and Analysis

**Figure 3:**
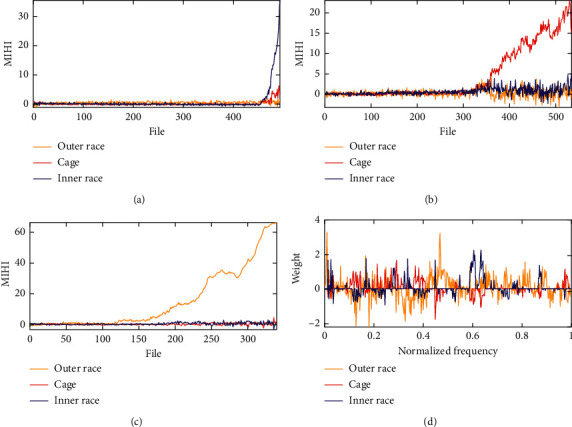
Results of degradation monitoring and weight of the component. (a) Inner race fault. (b) Cage fault. (c) Outer race fault. (d) Weight matrix (blue inner race, red cage, and yellow outer race).

Figure 3 shows the performance degradation assessment of MIHI for the different bearing fault monitoring. Here, the balance parameter *α* is set to 1, *n*_nor_ is set to 10, *β* is 2*e*^−4^, and *γ*=2*e*^−5^. The blue line denotes the condition monitoring health index of the inner race fault, the red line represents the cage fault monitoring, and the green color is the degradation trend of the outer race fault. To facilitate the observation, the MIHI value of each fault type monitor curve will minus the average of the first 50 files' MIHI value.

**Figure 4:**
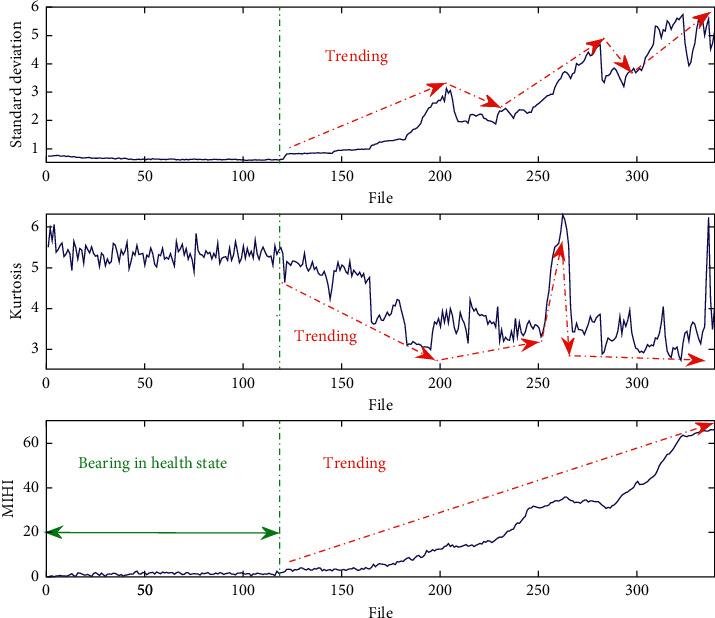
Bearing health state monitoring with different health indexes. (a) Standard deviation. (b) Kurtosis. (c) MIHI.

Figure 4 shows time-domain features that can indicate an occurrence of an incipient-bearing fault. To monitor the bearing health state, some time-domain features are used to quantify the bearing run-to-failure data, such as standard deviation and kurtosis. In Figure 4, we use the dataset bearing 2_5 to illustrate the superiority of the MIHI for bearing health monitoring. However, the time-domain features do not have monotonic trending, which is not beneﬁcial to the assessment of bearing degradation performance and prognostics. Meanwhile, the MIHI monitoring curve not only has a strong monotonic trending line but also can indicate an incipient-bearing fault.

To illustrate further the advantages of the proposed method, the natural variability of the proposed MIHI and how it is used for fault detection and incipient fault diagnosis are also provided. First, the MIHI at observation epochs 1–50 in a normal stage is used as a historical normal dataset. Second, whether the normal state dataset obeys the Gaussian distribution is checked. At a signiﬁcance level of 5%, the MIHI normal state datasets from bearings 2_1, 2_3, and 2_5 all satisfy the normal distribution conditions. Therefore, the Gaussian distribution assumption of the normal stage is accepted. Lastly, the three-sigma rule is used to detect a bearing abnormality, and the statistical threshold can be used as an early warning baseline for fault detection and beginning of degradation assessment.

**Figure 5:**
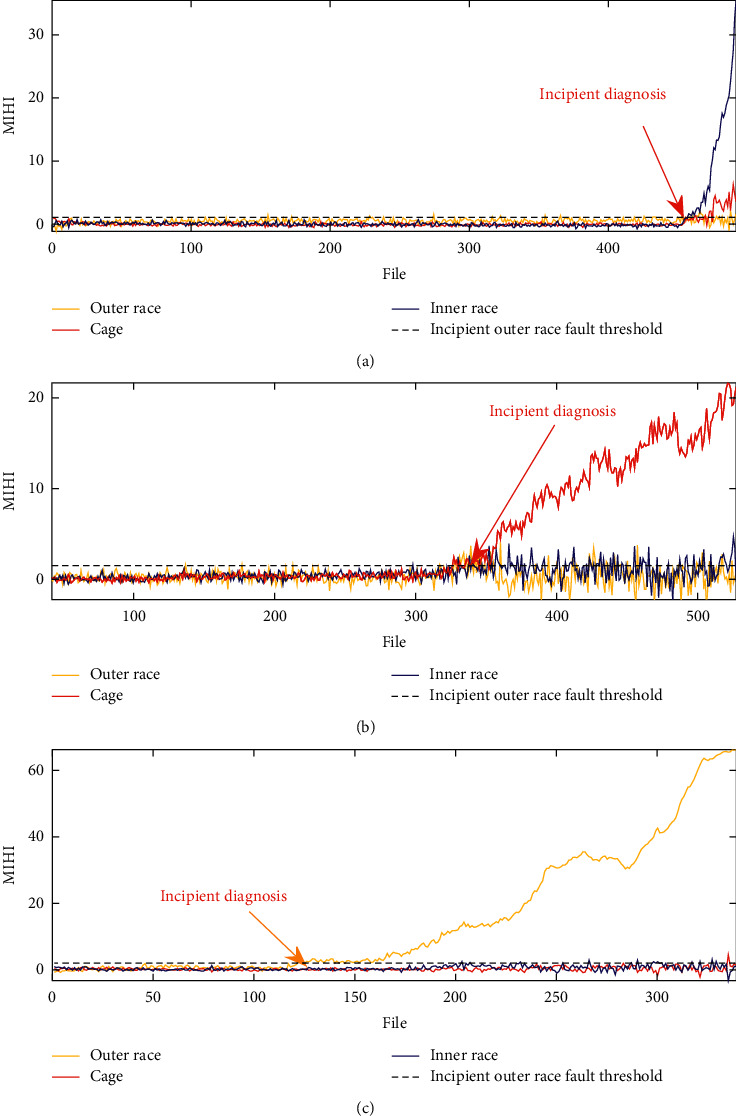
Bearing health state monitoring with proposed MIHI. (a) Inner race fault degradation. (b) Cage fault degradation. (c) Outer race fault degradation.

The proposed MIHI monitoring curve family and its corresponding incipient fault threshold are plotted in Figure 5. Combined with the statistical threshold, the proposed MIHI can realize bearing incipient fault diagnosis and continuous detection of the bearing degradation process.

### 4.3. Hyperparameter Analysis

The main hyperparameters of the proposed algorithm *α*, *β*, *γ*, and *n*_nor_ measure monotonicity, orthogonality, weight sparsity, and weight difference sparsity. These four hyperparameters are empirically chosen. In this section, the hyperparameter selection suggestion and the hyperparameters' effect on the final HIMI are studied.

First, a function is built to evaluate the MIHI condition monitoring curve's monotonicity:

(17) $$\begin{array}{c} \text{Monotonicity}=\overset{k}{\underset{j=1}{\sum }}\frac{\sum_{i=2}^{n_{j}}\left|\text{sign}\left({\text{HIMI}}_{i,j}^{j}-{\text{HIMI}}_{i,j}^{j}\right)\right|}{n_{j}-1}, \end{array}$$

where HIMI_*i*,*j*_^*j*^ means the *j*th value of *i*th observation **HIMI**_*i*_^*j*^ ∈ *ℝ*^*k*×1^, which is calculated by **HIMI**_*i*_^*j*^=**W** × **f**_*j*,*i*_. The larger the value of monotonicity, the better monotonicity the MIHI curve has.

Second, to indicate the orthogonality of the MIHI, a formula is utilized to calculate orthogonality:

(18) $$\begin{array}{c} \text{Orthogonality}=\overset{k}{\underset{j=1}{\sum }}\frac{\sum_{u=1}^{k}\text{std}\left(HIMI_{u,:}^{j}\right)-\text{std}\left(HIMI_{j,:}^{j}\right)}{\left|\max\left(HIMI_{j,:}^{j}\right)-\min\left(HIMI_{j,:}^{j}\right)\right|}, \end{array}$$

where **HIMI**^*j*^ ∈ *ℝ*^*k*×*n*_*j*_^, which is calculated by **HIMI**^*j*^=**W** × [**f**_*j*,1_, **f**_*j*,2_,…, **f**_*j*,*n*_*j*__], and **HIMI**_*j*,:_^*j*^ denotes the *j*th row of **HIMI**^*j*^. The smaller the value of orthogonality, the better orthogonality the MIHI curve family has.

In this illustrative example, the final fault type number is 3 (inner race, cage, and outer race); thus, *k*=3. Assume that the first fault degradation dataset is bearing 2_1 in Table 1 and consists of 491 files; thus, *n*_1_=491. The spectrum from the first file's signal in bearings 2_1, 2_3, and 2_5 is **f**_1,1_, **f**_2,1_, and **f**_3,1_, respectively. Our experiments show that even though the hyperparameter *n*_nor_(*n*_nor_=10) is set as a small value, the quasi-orthogonal sparse project algorithm can still help the MIHI curve family to obtain good orthogonality. For the rest of this section, the hyperparameter *n*_nor_ selection is not studied.

**Figure 6:**
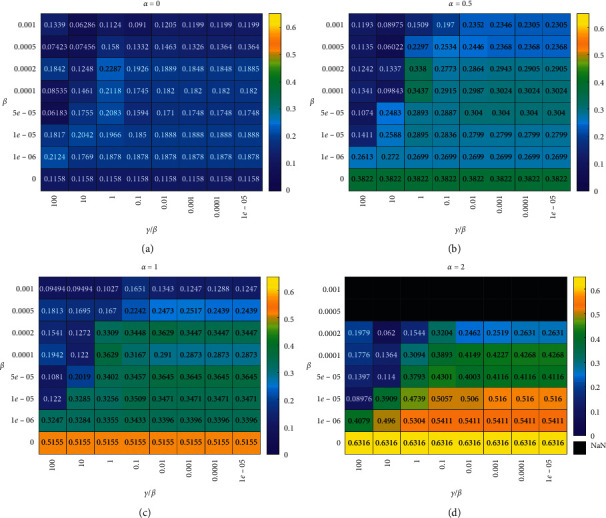
Monotonicity heatmap of hyperparameters *α*, *β*, and *γ*. (a) *α*  = 0, (b) *α*  = 0.5, (c) *α*  = 1, and (d) *α*  = 2.

**Figure 7:**
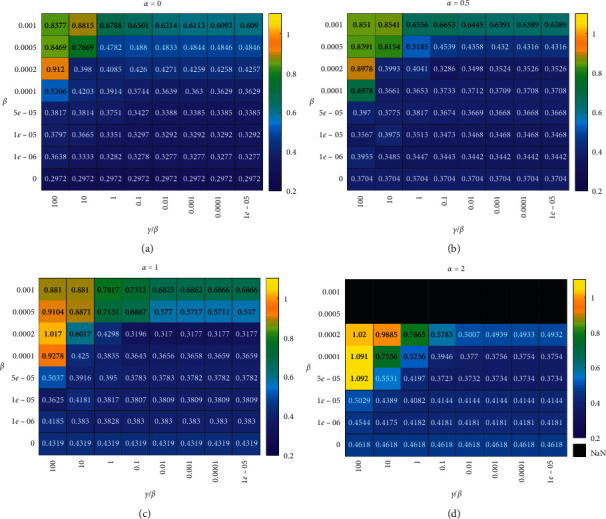
Orthogonality heatmap of hyperparameters *α*, *β*, and *γ*. (a) *α*  = 0, (b) *α*  = 0.5, (c) *α*  = 1, and (d) *α*  = 2.

The resulting heatmap of monotonicity is shown in Figure 6, and that of orthogonality is shown in Figure 7. We study four *α* values, i.e., 0, 0.4, 1, and 2. In each heatmap, we study eight *β* and *γ* values, which make up 64 combinations. Figures 6 and 7 not only indicate the hyperparameters' (*α*, *β*, and *γ*) effect on the monotonicity and orthogonality of the MIHI monitoring curve family but also offer the selection reference of hyperparameters *α*, *β*, and *γ*.

The hyperparameter *α* decides the MIHI monitoring curve's monotonicity. As shown in Figure 6, the bigger the parameter *α*, the more monotonic the MIHI monitoring curve is. However, Figure 7 indicates that the big *α* causes the MIHI monitoring curve family's orthogonality to decrease. Moreover, from all the heatmaps in Figures 6 and 7, *α*=1.

When prerequirement Properties 1–3 of the HIMI curve family are met, the sparser the weight matrix **W** is, the better overfitting is avoided. The heatmaps in Figures 6 and 7 show that *β* should be smaller than 0.0002 and *γ*/*β* should be smaller than 0.1; otherwise, the weight matrix **W** is too sparse to map the spectrum features into a MIHI monitoring curve family, which has good monotonicity and orthogonality.

**Figure 8:**
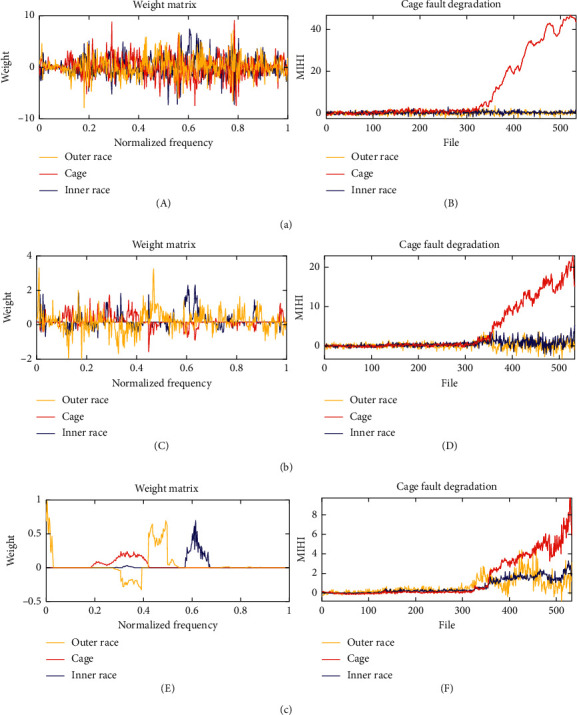
MIHI example of hyperparameters' change; the hypermeters *α*=1 and *n*_nor_=10 are the same, but *β* and *γ* are different; (a) *β*=0 and *γ*=0. (b) *β*=2*e*^−4^ and *γ*=2*e*^−5^. (c) *β*=5*e*^−4^ and *γ*=5*e*^−3^.

When the quasi-orthogonal sparse project algorithm only focuses on the first three properties: monotonicity, sensitivity, and orthogonality (*β* and *γ*=0), the MIHI monitoring curve family has the best monotonicity and orthogonality. However, the weight matrix is severely overfitted. The degradation process of bearing 2_3 is used to illustrate the effect of hyperparameters *β* and *γ* in Figure 8. Compared with the MIHI curve family when *β*=2*e*^−4^ and *γ*=2*e*^−5^, as shown in Figure 8(b), the monotonicity and orthogonality of the MIHI curve family when *β* and *γ*=0are stronger (Figure 8(a)), and the weight matrix fits all spectrum components. However, bearing fault characteristic information is not distributed in all frequency bands, and the weight matrix should only focus on the spectrum components related to the fault. The weight matrix calculated when *β* and *γ*=0 also fits with substantial noise, thus reducing the generality of MIHI. As shown in Figure 8(c), if the weight matrix is too sparse, then the MIHI monitoring curve family cannot satisfy Properties 1, 2, and 3. Thus, we recommend using hyperparameter heatmaps to select suitable hyperparameters when applying the proposed method.

## 5. Conclusions

This study proposed a method of building a MIHI to trace the various bearing degradation trends with various types of faults. The proposed method is a linear transform algorithm that maps high-dimensional observation features into low-dimension MIHI, indicating the bearing's various fault type degradation trends at the same time. Meanwhile, inspired by the orthogonal vector, we proposed utilizing orthogonality to develop the health index of bearing degradation trend monitoring. This algorithm also introduces weight sparsity and weight difference sparsity to avoid overfitting. The proposed algorithm has explicit and simple mathematical expressions, and the process of calculation does not rely on the complex optimization algorithm. Therefore, the method is suitable to deal with situations that have high-dimensional observation features.

## Figures and Tables

**Algorithm 1:**
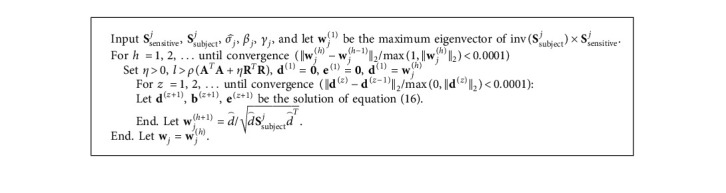
Algorithm 1.

**Table 1:** Detail of the illustrative example.

Condition	Bearing no.	Time duration	Number of measurement files	Final fault
Speed: 37.5 Hz	Bearing 2_1	8 h 11 min	491	Inner race
Load: 11 kN	Bearing 2_3	8 h 53 min	533	Cage
	Bearing 2_5	5 h 39 min	339	Outer rage

## Data Availability

The data used to support the findings of this study are included within the article.

## Nomenclature

*k*: Number of fault types
*m*: The dimension of the spectrum feature
*α*: MIHI balance parameter
*β*: MIHI sparsity penalty parameter
*γ*: MIHI weight difference sparsity penalty parameter
*n*_nor_: Number of the epoch of bearing normal state
*η*: Lagrangian penalty parameter when solving
*l*: Approximation parameter when solving
*w*_*j*,*q*_: *j* − row and *q* − column value of **W**
**W** ∈ *ℝ*^*k*×*m*^: Project matrix of MIHI
**w**_*j*_ ∈ *ℝ*^1×*m*^: *j* row of **W**
**F**_*j*_ ∈ *ℝ*^*m*×*n*_*j*_^: Spectrum feature matrix of *j*th fault type degradation process data
**f**_*j*,*i*_ ∈ *ℝ*^*m*×1^: Spectrum feature of *j*th fault type *i*th observation epoch
**N**_*j*_ ∈ *ℝ*^*m*×*n*_nor_^: Normal state observation matrix of *j*th fault type
**μ**_*j*_ ∈ *ℝ*^*m*×*1*^: Mean vector of **N**_*j*_
$\hat{\sigma_{j}}\in {\mathbb{R}}^{1\times m}$: Project matrix *j* − row's feature penalty vector
**A** ∈ *ℝ*^*m*×*m*^, **b** ∈ *ℝ*^1×(*m* − 1)^, **c** ∈ *ℝ*^*m*×1^, **d** ∈ *ℝ*^1×*m*^, **e** ∈ *ℝ*^1×(*m* − 1)^, **Q** ∈ *ℝ*^*m*×*m*^, **G** ∈ *ℝ*^*m*×*m*^: Intermediate variables in solving process
